# The PPP1R15 Family of eIF2-alpha Phosphatase Targeting Subunits (GADD34 and CReP)

**DOI:** 10.3390/ijms242417321

**Published:** 2023-12-10

**Authors:** Danielle Hicks, Krithika Giresh, Lisa A. Wrischnik, Douglas C. Weiser

**Affiliations:** 1 Department of Science, Mathematics and Engineering, Modesto Junior College, Modesto, CA 95350, USA; 2 Department of Biological Sciences, University of the Pacific, Stockton, CA 95211, USA

**Keywords:** GADD34, CReP, PPP1R15, PPP1R15A, PPP1R15B, PP1, PPP1CA, unfolded protein response (UPR), eIF2α, integrated stress response (ISR)

## Abstract

The vertebrate PPP1R15 family consists of the proteins GADD34 (growth arrest and DNA damage-inducible protein 34, the product of the PPP1R15A gene) and CReP (constitutive repressor of eIF2α phosphorylation, the product of the PPP1R15B gene), both of which function as targeting/regulatory subunits for protein phosphatase 1 (PP1) by regulating subcellular localization, modulating substrate specificity and assembling complexes with target proteins. The primary cellular function of these proteins is to facilitate the dephosphorylation of eukaryotic initiation factor 2-alpha (eIF2α) by PP1 during cell stress. In this review, we will provide a comprehensive overview of the cellular function, biochemistry and pharmacology of GADD34 and CReP, starting with a brief introduction of eIF2α phosphorylation via the integrated protein response (ISR). We discuss the roles GADD34 and CReP play as feedback inhibitors of the unfolded protein response (UPR) and highlight the critical function they serve as inhibitors of the PERK-dependent branch, which is particularly important since it can mediate cell survival or cell death, depending on how long the stressful stimuli lasts, and GADD34 and CReP play key roles in fine-tuning this cellular decision. We briefly discuss the roles of GADD34 and CReP homologs in model systems and then focus on what we have learned about their function from knockout mice and human patients, followed by a brief review of several diseases in which GADD34 and CReP have been implicated, including cancer, diabetes and especially neurodegenerative disease. Because of the potential importance of GADD34 and CReP in aspects of human health and disease, we will discuss several pharmacological inhibitors of GADD34 and/or CReP that show promise as treatments and the controversies as to their mechanism of action. This review will finish with a discussion of the biochemical properties of GADD34 and CReP, their regulation and the additional interacting partners that may provide insight into the roles these proteins may play in other cellular pathways. We will conclude with a brief outline of critical areas for future study.

## 1. Introduction

**Figure 1:**
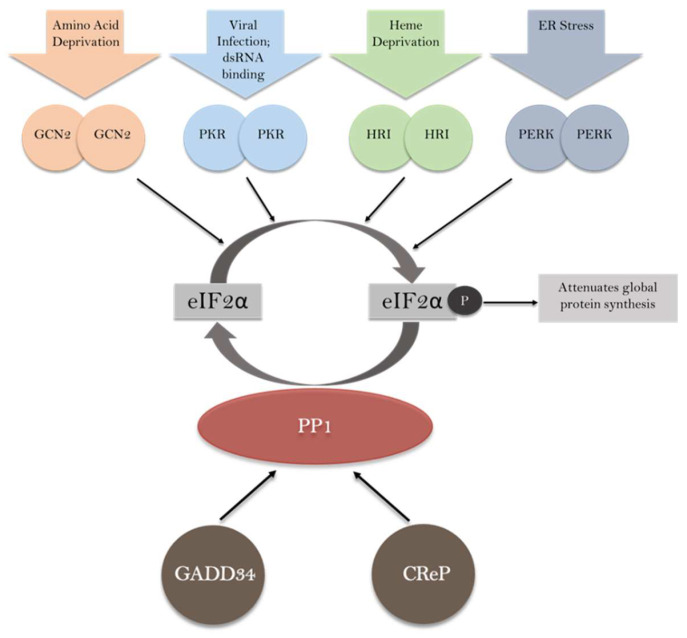
Reversible phosphorylation of eIF2α regulates global protein synthesis and the translation of specific mRNAs. Phosphorylation of eIF2α by a family of four kinases, GCN2, PKR, HRI and PERK, is induced by a diverse array of cellular stimuli and stressors. Phosphorylation of eIF2α attenuates the translation of most mRNAs. GADD34 and CReP bind to PP1 and promote dephosphorylation of eIF2α.

The primary function of the GADD34 and CReP proteins is the regulation of protein phosphatase 1 (PP1), which can modulate the activity of the eukaryotic initiation factor 2 alpha (eIF2α) subunit. The combined function of eIF2α kinases and PP1 phosphatase to restore cellular homeostasis following a diverse array of cellular stresses is termed the integrated stress response (ISR, Figure 1) [1,2]. Protein phosphatase 1 (PP1) is an abundant and ubiquitously expressed eukaryotic serine–threonine phosphatase that regulates diverse cellular processes including actomyosin contractility, glycogen metabolism, cell cycle progression, gene expression, protein synthesis and neuronal signaling [3,4,5,6,7]. The purified PP1 catalytic subunit is highly promiscuous and will dephosphorylate an array of phosphoproteins [3,4,5,6,7]. In the cell, however, a suite of regulatory and inhibitory subunits precisely controls PP1 activity [3,5,7,8]. Regulatory or targeting subunits bind to PP1 and can target PP1 to a subset of substrates, inhibit PP1 binding to other “off-target” substrates, control PP1 subcellular localization and bring the assembled holoenzyme form of PP1 under the control of various upstream signaling pathways [3,5,8,9]. Most PP1 inhibitory subunits directly compete with regulatory subunits for PP1 binding and are much more potent against the isolated catalytic subunit than holoenzyme forms [10]. Some PP1 inhibitors form tripartite complexes with PP1 and regulators, allowing them to effectively inhibit holoenzymes [11,12,13,14,15]. Many organisms have multiple genes for PP1; the protein products are referred to as PP1 isoforms, which have similar but not identical functions [16,17,18,19]. Interestingly, all three mammalian genes (PP1α, PP1β and PP1γ) appear to have nearly ubiquitous expression in all organisms examined [20], and their amino acid sequences have diverged very little, although a few important splice variants have been observed [21]. 

This review will focus on two members of the PPP1R15 family of PP1 targeting subunits, GADD34 and CReP. The primary cellular function of both proteins is to bind to PP1 and to direct the dephosphorylation of the eukaryotic translation initiation factor eIF2α [22,23,24]. eIF2α associates with eIF2β and eIF2γ, forming the eIF2 complex [25]. The eIF2 complex assembles Met-tRNA with the small and large ribosomal subunits at the AUG on the mRNA, forming the translation initiation complex in a GTP-dependent manner. The phosphorylation of serine-51 on eIF2α inhibits GDP–GTP exchange, which is required for another round of translation initiation [26,27]. Thus, the phosphorylation of eIF2α attenuates global protein translation [25]. 

The phosphorylation of eIF2α is mediated by a number of kinases and is a major mechanism of translation control in cells experiencing environmental or metabolic stress [2]. Mammals express four different eIF2α kinases (Figure 1), each of which monitors for different exogenous and endogenous stresses [27]. The four kinases are general control nonderepressible (GCN2, or EIF2AK4), which is induced in response to nutritional stresses and amino acid deprivation; PKR-like endoplasmic reticulum kinase (PERK, or EIF2AK3/PEK), which is induced in responses to ER stress; heme-regulated inhibitor (HRI, or EIF2AK1), which is activated by heme deprivation in red blood cells; and protein kinase R (PKR, or EIF2AK2), which participates in an antiviral defense pathway involving interferons [26]. Even though the phosphorylation of eIF2α leads to the attenuation of global protein synthesis, certain mRNAs that encode stress-related proteins are selectively translated [28]. One such protein is ATF4, whose mRNA contains a short, upstream open-reading frame (uORFs) within its 5′ translated region [29,30,31]. In ATF4, these uORFs are inhibitory toward translational initiation by promoting translational termination prior to translating the CDS [30]. When eIF2α is phosphorylated, ribosome bypass or leaky scanning through inhibitory uORFs allows for the translation of ATF4 [30]. The upregulation of ATF4 promotes the synthesis of multiple prosurvival genes [31]. 

Prolonged phosphorylation of eIF2α also allows for efficient translation using unconventional modes of translation initiation, including through eIF2A [32]. The alternative translation initiation factor eIF2A is a monomer that is functionally distinct from the trimeric eIF2 complex [32]. The role of eIF2A in global translation is limited [33], but it can play an important role in internal initiation, reinitiation and non-AUG initiation [32]. For example, during conditions of prolonged eIF2α phosphorylation, the high-level expression of ER chaperone BiP requires eIF2A-mediated translational initiation at non-AUG start codons [34]. Initiation at non-AUG codons is critical for tumor initiation and progression [35] and for tumor cells to survive the stress of paclitaxel treatment [36]. In addition, eIF2A is critical for the continued efficient translation of C9ORF72 in neurons with active ISR [37]. Repeat expansion in the C9ORF72 gene is critical for amyotrophic lateral sclerosis (ALS) and frontotemporal dementia (FTD) (reviewed in [38]). 

## 2. The Role of eIF2α Phosphatases in Cellular Stress Responses

### 2.1. The Unfolded Protein Response

**Figure 2:**
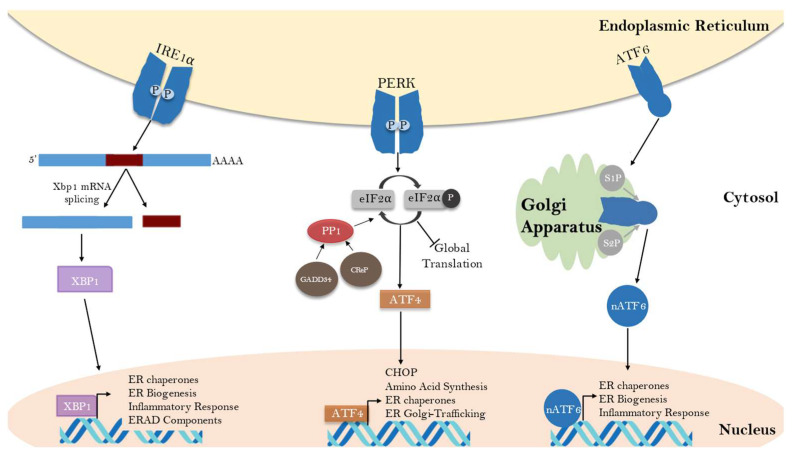
The three branches of the unfolded protein response (UPR). The three branches of the UPR (IRE1α, ATF6 and PERK) are activated in response to the accumulation of unfolded proteins in the ER lumen leading to a complex transcriptional and translational cascade. Activated IRE1α leads to the unconventional splicing of the Xbp1 mRNA. Only this spliced mRNA encodes for the transcription factor XBP1. PERK activation leads to the phosphorylation of eIF2α, translational inhibition and activation of the transcription factors ATF4 and CHOP. Stress-induced proteolytic cleavage of the transcription factor ATF6 produces an active fragment called nATF6, which translocates from the ER to the nucleus. All three pathways produce a transcriptional response which serves to increase the protein-folding capacity of the ER. This is aided by PERK-mediated global translational inhibition and IRE1-mediated degradation of ER-bound mRNAs. In addition to this adaptive response, the UPR can activate inflammatory and apoptotic pathways.

Although they are likely involved in multiple aspects of the ISR, the pathway in which GADD34 and CReP have been most implicated is the unfolded protein response (UPR), in which ER stress activates signal transduction pathways that cause a complex, multibranched response in transcription and translation (Figure 2). In all eukaryotic cells, the endoplasmic reticulum (ER) serves the important roles of protein translation and folding and the processing of all secretory and membrane-bound proteins, as well as calcium storage [39]. The ER processes approximately one-third of the cell’s proteins, so cells must regulate protein homeostasis in the ER, termed proteostasis, by controlling and balancing protein synthesis, assembly, trafficking, folding and degradation [40]. The ER must maintain a high level of quality and efficiency in protein folding for cells to survive and function normally, but because of the complex and intricate nature of protein folding, it is the most error-prone step in the expression of any particular gene [40,41]. Inside the ER, protein folding is facilitated by resident chaperones, which are enzymes that catalyze protein-folding reactions or stabilize proteins in a competent state for folding [42,43]. Proteins that are correctly folded and modified into their mature conformation are permitted to move through the secretory pathway; they are transported to the Golgi apparatus, where they are either sorted to be sent to various cellular compartments, recycled back to the ER or secreted into extracellular space [44]. 

ER stress occurs when the load of proteins to be folded in the ER exceeds the chaperone-mediated folding capacity to accurately process proteins, resulting in an increased accumulation of misfolded proteins in the ER lumen [45]. Protein misfolding negatively impacts cell function because the necessary proteins are not able to leave the ER and perform their normal functions [46]. Overall, the UPR leads to increased chaperone activity, decreased protein synthesis, and increased protein degradation by ER-associated protein degradation (ERAD). ER stress can be caused by a variety of intrinsic and extrinsic factors, including increased levels of protein synthesis, impaired ubiquitination and proteasomal degradation, deficient autophagy, energy deprivation, excessive or limited nutrients, dysregulated calcium levels or redox homeostasis, inflammation and hypoxia [40].

Three UPR signaling pathways have been characterized that sense and respond to ER stress (Figure 2). The most evolutionarily conserved is the IRE1–XBP1 pathway (inositol-requiring protein 1α–X-box-binding protein 1) [47]. In this branch of the UPR, when the load of unfolded proteins in the ER lumen exceeds ER folding capacity, the protein-folding chaperone BiP (binding immunoglobulin protein) detaches from IRE1, which is a trans-ER membrane-bound protein [48]. BiP detachment results in trans-autophosphorylation of the IRE1 homodimer, activating IRE1 endonuclease activity [49]. IRE1 can then unconventionally splice XBP1 mRNA, altering the reading frame for XBP1 translation, which results in the synthesis of a transcription factor. The XBP1 transcription factor activates target genes that encode protein chaperones and components of ERAD [50,51]. IRE1α also cleaves some mRNAs that contain XBP1-like secondary structures to contribute to mRNA degradation in a process known as regulated IRE1α-dependent decay (RIDD) [52]. In this process, mRNAs encoding transmembrane and secretory proteins are targeted to reduce ER stress by decreasing the incoming protein load.

The next UPR pathway upregulates activating transcription factor ATF6. The ATF6 protein is also a trans-ER membrane protein, and upon BiP dissociation, it is translocated to the Golgi apparatus, where it is cleaved to become an active transcription factor [53,54]. ATF6α then enters the nucleus to activate the transcription of various genes involved in ER stress recovery (including the XBP1 gene), several genes encoding protein chaperones (such as BiP, ER protein 57 (Erp57), glucose-regulated protein (GRP94) and genes for ER-associated protein degradation functions [55,56]. 

The third pathway of the UPR is mediated by PKR-like ER kinase (PERK), a component of the ISR, which attenuates global protein translation to decrease the rate of proteins entering the ER via eIF2α phosphorylation. The global downregulation of protein synthesis conserves cellular resources and provides time to evaluate the severity of the protein misfolding and then reprograms gene expression either toward a prosurvival repair response or towards apoptosis when ER stress is continuous or severe [57]. PERK is an ER-resident transmembrane kinase that is inactive during unstressed conditions through its association with BiP [48]. Upon BiP dissociation, PERK oligomerizes, autophosphorylates and then phosphorylates the α subunit of eIF2 at serine 51 [58]. The phosphorylation of eIF2α inhibits the activity of the guanine nucleotide exchange factor eIF2B, which is required for the formation of eukaryotic translational preinitiation complexes [24,59]. This transient reduction in the overall amount of proteins in the ER gives stressed cells an opportunity to clear misfolded proteins from the ER and protects cells against the potential toxicity of misfolded proteins [59,60]. 

Although the phosphorylation of eIF2α acts to attenuate global protein synthesis, it selectively allows for more efficient translation of mRNAs that encode for stress-related proteins [61,62]. During the UPR, certain mRNA transcripts show favored translation in the state of globally reduced functional translation initiation complexes, typically those with short upstream open-reading frames (uORFs) in their 5’ untranslated region [57]. One such protein is activating transcription factor 4 (ATF4), which is upregulated after eIF2α phosphorylation and both increases the capacity for protein transport in the ER and promotes the synthesis of multiple prosurvival genes [32]. ATF4 enters the nucleus to activate UPR genes encoding proteins necessary for autophagy, antioxidant response, amino acid biosynthesis and transport, protein chaperones and the promotion of ERAD, which are all hallmarks of the early UPR response [63]. ATF4 activates the transcription of GADD34, which serves to promote eIF2α dephosphorylation and restore global protein translation [22]. ATF4 also promotes the transcription of CHOP (CCAAT/enhancer-binding protein “C/EBP” homologous protein) by binding to and activating the CHOP promoter [22,32,64,65,66]. CHOP transcription is strongly induced by ER stress, and the CHOP mRNA, which contains uORFs, is translationally upregulated by the phosphorylation of eIF2α [67,68]. While CHOP has some cytoprotective targets, prolonged expression, especially in the presence of sustained PERK activation, can trigger the expression of proapoptotic genes [66]. The PERK signaling pathway is therefore capable of promoting both an adaptive, prosurvival response and an apoptotic response to ER stress. The severity and context of the ER stress, duration of UPR signaling and cell type and environment are all factors that contribute to the outcome of PERK signaling. 

**Figure 3:**
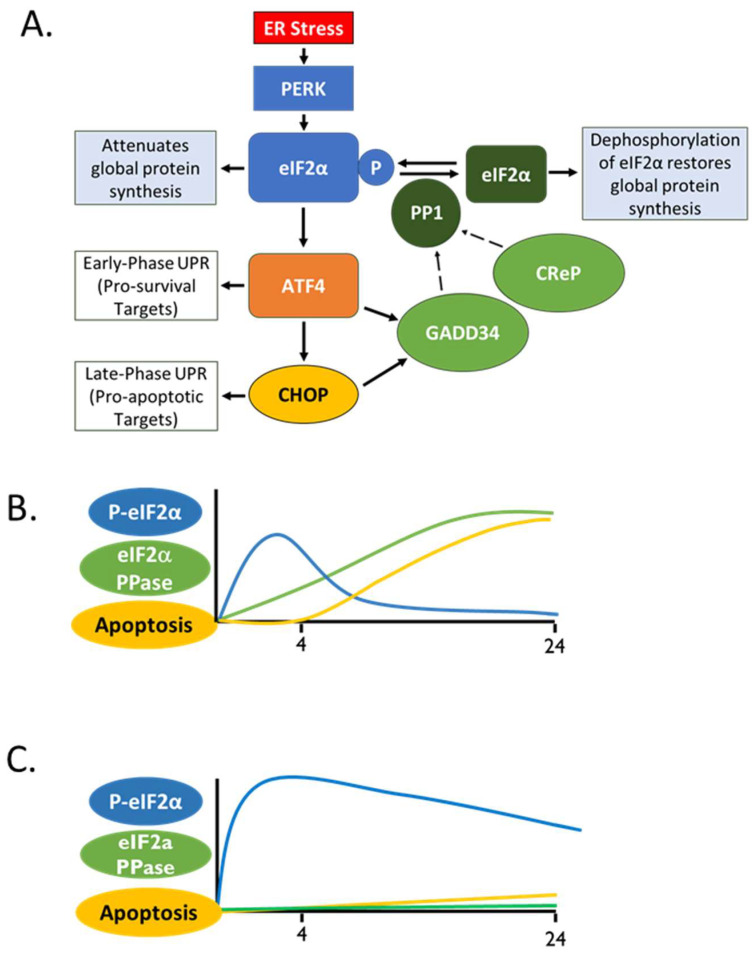
GADD34 and CReP coordinate feedback inhibition of the PERK pathway, which governs a switch from protective to apoptotic cellular responses. (**A**) In response to ER stress, the phosphorylation of eIF2α by PERK results in global protein translation attenuation and the upregulation of a prosurvival gene cascade through ATF4 activation. In the case of prolonged stress and sustained eIF2α phosphorylation, ATF4 and CHOP can lead to a proapoptotic pathway in conditions of prolonged ER stress. Both ATF4 and CHOP can activate transcription of GADD34. GADD34 and CReP are two targeting subunits that interact with PP1 to dephosphorylate eIF2α, with GADD34 being a potent feedback inhibitor of PERK signaling. (**B**) Prolonged stress leads to the accumulation of eIF2α phosphatase activity, resulting in dephosphorylation of eIF2α and a restoration of translation. This allows for the efficient translation of proapoptotic genes downstream of CHOP signals. (**C**). If eIF2α phosphatase activity is reduced through inhibition of GADD34 and/or CReP, the early phase of the UPR is prolonged and CHOP-dependent apoptotic signals are attenuated.

As feedback inhibitors of the PERK pathway, GADD34 and CReP play a critical role in regulating the progression of the UPR from a distinct early stage into the late stage (Figure 3) [28,69]. The early stage occurs over the initial 30 min to 1 h of ER stress and is marked by translation suppression and degradation of misfolded proteins, as well as low expression levels of both GADD34 and CReP and high levels of eIF2α phosphorylation. The later stage, beginning around 4 h post ER stress, focuses on enhancing ER protein folding capacity by increasing the synthesis of chaperones [62]. Around 4 h after stress, GADD34 functions to progress the UPR. At this point, GADD34 levels have increased considerably, and this is essential for the return of the mRNAs encoding ER-targeted proteins that are released from the ER 30 min after stress, and cells show nearly full recovery of protein synthesis after 8 h [28]. Cells lacking GADD34 can partially compensate by upregulating CReP, though cell recovery is delayed [28]. GADD34 mutant cells show elevated eIF2α phosphorylation at 8 h after ER stress induction, and this phosphorylation slowly declines at around 18–24 h post stress induction [28]. This prolonged elevation of eIF2α phosphorylation suppresses protein synthesis for much longer than normal, only showing a moderate increase at 24 h [28]. Prolonged elevation of phosphorylated AKT, an inhibitor of PERK phosphorylation and activation, also seems to contribute to this partial recovery of protein synthesis when GADD34 is not present [28]. Following prolonged activation, the PERK pathway leads to cell death through CHOP-dependent mechanisms after the dephosphorylation of eIF2α [66] (Figure 3B). If eIF2α dephosphorylation is inhibited, however, CHOP induction is attenuated and proapoptotic signals are inhibited (Figure 3C) [70,71,72]. The prolonged global translational inhibition caused by the inhibition of eIF2α dephosphorylation also results in the less efficient translation of proapoptotic “late UPR genes” and prolonged translation of prosurvival “early UPR genes” [28,69]. The delay in the progression of UPR signaling with GADD34 inhibition may explain the neuroprotective effects of the pharmacological inhibitors discussed later in this review.

The upregulation of CHOP occurs after an accumulation of ATF4 via the activation of the PERK pathway. Although CHOP accumulation correlates with apoptosis, both ATF4 and CHOP mRNAs and proteins have short half-lives, so strong and chronic PERK activation is necessary to promote high levels of CHOP [40]. Also, ER stress is capable of activating apoptosis in a CHOP-independent manner through Puma and p63 [73]. The human CHOP protein contains transcriptional activation/repression domains at its N-terminus and a C-terminal basic–leucine zipper (bZIP) domain containing a DNA-binding region followed by a leucine zipper motif for dimerization [74,75]. CHOP’s bZIP domain allows it to form heterodimers with other C/EBPs and another bZIP group, the CREB/ATF family, and it has been reported to be required for processes in CHOP-induced apoptosis [76,77]. The precise mechanism(s) by which CHOP initiates its apoptotic response is not fully known, but many studies have found various ways in which CHOP sensitizes ER-stressed cells to apoptosis [78]. CHOP plays a role in inducing GADD34 expression, potentially through direct binding to the GADD34 promoter [66]. Through GADD34 activation, CHOP promotes the reversal of the translational repression initiated by the PERK pathway [79]. Increased protein synthesis can contribute to the ER stress of the cell and predispose cells to CHOP-mediated apoptosis in conditions of continuous or severe ER stress [80]. CHOP is involved in significantly disrupting redox homeostasis, which prepares stressed cells for apoptosis [81]. CHOP has been found to deplete cells of glutathione, an important intracellular scavenger of ROS [81], and a direct target gene of CHOP, ERO1α, promotes oxidizing conditions in the ER and an accumulation of reactive oxygen species, contributing to protein misfolding and death in stressed cells [66]. CHOP has also been found to sensitize cells to ER stress-induced apoptosis by inhibiting the transcription of the antiapoptotic protein Bcl2, upregulating the proapoptotic protein Bim, promoting translocation of the proapoptotic effector protein Bax from the cytosol to the mitochondria and targeting CDK2 and CDK4 for degradation to cause cell growth arrest [81]. Interestingly, transcriptomic and CHIP-seq experiments in mouse embryonic fibroblasts did not find CHOP or ATF4 occupation of the promoters of any proapoptotic genes [80]. Rather, ATF4 and CHOP were seen to form heterodimers that upregulate genes involved in the UPR for functions like autophagy, mRNA translation, protein synthesis, ATP depletion and oxidative stress [80]. These data suggest that cell death is indirectly promoted by CHOP through its primary role of supporting the restoration of protein synthesis.

### 2.2. The Genetics of eIF2α Phosphatases

The critical role of PP1 in the dephosphorylation of eIF2α is highly evolutionarily conserved. In *Saccharomyces cerevisiae*, no orthologues of the PPP1R15 proteins have been found; instead, yeast relies on a PP1-binding element in a region of the eIF2γ subunit to target PP1 to dephosphorylate eIF2α [82]. Yeast eIF2γ has an N-terminal extension containing a PP1-binding motif (KKVAF or RVxF motif, a sequence found in many but not all PP1 binding proteins [83,84,85]) that allows eIF2γ to recruit GLC7 (the functional homolog of PP1 in yeast) and target it to dephosphorylate eIF2α [82]. Yeast eIF2γ does not share sequence homology with GADD34 or CReP, except from the conserved PP1-binding motif (RVxF), nor does the mammalian eIF2γ contain the unique N-terminal extension found in yeast [82]. The system of eIF2α dephosphorylation in yeast indicates that maintaining appropriate levels of eIF2α phosphorylation is necessary for cellular homeostasis to protect cells from prolonged eIF2α phosphorylation. Interestingly, the RVxF motif of eIF2γ is restricted to *Saccharomyces* and closely related organisms but is missing from other fungal model organisms such as *S. pombe* and *Aspergillus* [82]. The genomes of both the *S. pombe* and *Aspergillus* strains also lack homologs of CReP or GADD34. In addition, no CReP or GADD34 homolog has been identified in plants or in *C. elegans*, suggesting that additional proteins or mechanisms have evolved to control eIF2α dephosphorylation in these organisms.

In *Drosophila*, a single PPP1R15 protein (dPPP1R15) is responsible for eIF2α dephosphorylation, yet it shares characteristics with both mammalian eIF2α phosphatase-targeting subunits [86,87]. dPPP1R15 shares 59% sequence homology over a 61-residue stretch with both mammalian PPP1R15 proteins and also localizes to the ER. dPPP1R15 does not exhibit significant transcriptional regulation in response to ER stress but is translationally upregulated [87]. Due to its constitutive expression and close ER association, dPPP1R15 is functionally homologous to mammalian PPP1R15b [87]. PPP1R15 loss-of-function studies reveal the importance of eIF2α dephosphorylation; in *Drosophila*, the total depletion of dPPP1R15 leads to significant embryonic lethality, and partial depletion leads to severe developmental delay [87].

The genetic knockout of GADD34 and CReP in mice revealed the primary function of these eIF2α phosphatases and the resulting phenotypes in mammalian physiology [88,89,90,91]. Mutant mice homozygous for a knockout allele of the GADD34 gene survived to adulthood and were fertile, with the only defects detectable upon treatment with an ER or physiological stressor [88]. Mutant mice homozygous for a knockout allele of CReP were about half the size of WT at birth, pale in color and failed to nurse, with none surviving past the first day of postnatal life [88]. The pallor was explained by low hematocrit, with abnormalities in red cell size and shape. The livers showed compensatory proliferation in blood precursors, implicating an important role for CReP in fetal erythropoiesis. Mutating serine 51 on eIF2α was sufficient to rescue the CReP mutant mice’s size and red blood cell counts [88]. Liver cytoplasmic extracts also showed elevated GADD34, indicating compensation for CReP deficiency. Embryos that lacked all PPP1R15 function, failed to form a blastocyst cavity, grow or hatch from the zona pellucida and did not develop past the preimplantation period. This is likely due to a failure to deal with the significant increase in protein synthesis that occurs at the 2–8 cell stage. In this stage, gene expression for GADD34 and CReP is activated, and genes that inhibit translation are repressed, such as PERK [88]. Again, double homozygous mutants lacking both GADD34 and CReP were able to be rescued by the serine-51 mutation on eIF2α. Together, these data indicate that efficient embryogenesis relies on the precise regulation of eIF2α phosphorylation [88].

In humans, a homozygous missense mutation in CReP (R658C) has been found to cause an autosomal recessive condition resulting in multisystem abnormalities: patients present with severe microcephaly, short stature, hypoplastic brainstem and cord, delayed myelination and intellectual disability, pancreatic β-cell failure and diabetes and bone deformities [92,93]. This mutation was located within the C-terminal PP1-binding domain, specifically changing the highly conserved arginine 658 to a cysteine [92]. This R658C mutation results in reduced interaction between CReP and PP1c, increased eIF2α phosphorylation and the sensitization of β-cells to apoptosis [92,93]. No human mutations in GADD34 have been characterized. In addition, mutations that decrease eIF2α phosphorylation, such as mutation of the kinase (EIF2AK3) in Wolcott–Rallison syndrome, are also deleterious to β-cells [94,95]. This indicates that β-cells are exquisitely sensitive to the dysregulation of eIF2α. These studies revealed that eIF2α dephosphorylation is the essential function of the PPP1R15 family but also highlighted distinct roles for GADD34 and CReP, with CReP being uniquely crucial for survival.

### 2.3. eIF2α Phosphatases in Health and Disease

Understanding UPR signaling and the specific roles of GADD34 and CReP has far-reaching applications in human disease. Numerous diseases arise from defects in protein folding, including several neurodegenerative diseases and metabolic diseases, while ER stress is also a consequence of other pathologies, such as cancer progression [96]. ER stress contributes to the initiation and progression of many human diseases, due to the fact that many factors involved in pathogenesis are also causes of ER stress. Because prolonged UPR activation can induce apoptosis, chronically stressed cells become more susceptible to ER stress-induced apoptosis, contributing further to disease phenotypes. Genetic mutations in genes involved in UPR signaling or in proteins processed by the ER are known to cause and contribute to disease.

Neurodegenerative disease progression and age-related diseases are associated with the accumulation of misfolded proteins in neurons and oxidative stress, which activates the ER stress-induced apoptosis of neuronal cells [97]. Neuron loss results in cognitive and neural deficits, as seen in diseases like Parkinson’s and Alzheimer’s. In human and mouse models of Alzheimer’s disease, elevated levels of phosphorylated eIF2α, ATF4 and IRE1 have been identified, confirming that UPR pathways are involved [98]. Specific misfolded proteins have been identified in several diseases, such as amyloid-β peptides in Alzheimer’s disease and α-synuclein in Parkinson’s disease. The abnormal accumulation and aggregation of such proteins induce ER stress and neuron loss and can even exert inhibitory effects on UPR signaling. For example, α-synuclein inhibits ATF6α processing directly through physical interactions and indirectly hinders protein transport from the ER to the Golgi [99]. Although the UPR is known to be involved in neurodegenerative diseases, specific mutations in UPR pathways that cause neurodegeneration in humans have not yet been identified [40].

ER homeostasis helps regulate glucose and lipid metabolism, so ER stress and UPR pathways can contribute to various metabolic diseases, such as diabetes and nonalcoholic fatty liver disease (NAFLD) [40]. ER stress causes lipids to accumulate in hepatocytes, and the three UPR pathways are essential for limiting and clearing this accumulation [100]. In type 2 diabetes, insulin resistance causes β-cells to increase insulin synthesis and secretion, making these cells more susceptible to ER stress-induced apoptosis. The precise balance of the PERK pathway is required for β-cells survival, and mutations in GADD34, CReP or PERK result in diabetes [93,94,95]. In type 1 diabetes, inflammation induces ER stress in β-cells, making insulin synthesis and secretion more difficult and also sensitizing cells to apoptosis. The hyperglycemia and hyperlipidemia associated with metabolic disease can induce ER stress in endothelial cells and neurons. GADD34 overexpression has been seen to protect against high-fat-diet-induced liver steatosis in mice by suppressing genes involved in lipogenesis [101].

ER stress and UPR activation have been found in a variety of human cancers (reviewed in [96]). Cancers often develop and progress in stressful microenvironment conditions, such as hypoxia, nutrient deprivation and poor vascularization, resulting in ER stress and consequent UPR activation to survive. Tumors may also use the UPR to survive increased demands in protein synthesis needed for oncogenic transformation, tumor growth or malignant progression. Since the PERK-eIF2α pathway of the UPR can either promote survival or apoptosis, the context of the cancer likely determines the impact of this pathway on tumor progression. Current studies support this paradoxical role of PERK in tumorigenesis. An antiproliferative role for the PERK pathway was found in normal mammary epithelial cells, where PERK was essential for responding to adhesion-regulated signals to prevent mammary tumor formation, and the inhibition of GADD34 was sufficient to halt the growth of ErbB2-positive tumors [102,103]. Conversely, a prosurvival function has also been supported for PERK-dependent signaling; in animal models of mammary carcinoma, PERK helps to maintain redox homeostasis and prevent the activation of the oxidative DNA damage checkpoint, facilitating tumor growth [104]. In extreme hypoxia, signaling by PERK-eIF2α-P promotes the tolerance of cancer cells to these conditions, and the inactivation of the PERK pathway can impair the survival of these cells [105,106]. Similarly, the role that CHOP plays in tumorigenesis is also divergent, although the predominant amount of evidence supports a proapoptotic role for CHOP-induced apoptosis in stress. For example, in a mouse model of K-rasG12V-driven lung cancer, CHOP appears to control the early stages of tumor progression by inducing apoptosis in response to microenvironmental stress [106]. However, some recent studies have indicated tumor-supporting functions of CHOP in certain cells. Myeloid-derived suppressor cells with immunosuppressive activity within tumors expressed CHOP without fully undergoing apoptosis, suggesting a potential benefit to inhibiting CHOP in certain cancers [107]. As the role of ER stress in diseases has become clearer, there has been increasing interest in studying the causes and effects of ER stress in a variety of human diseases, and this field is expanding rapidly, with a special focus on pharmacological agents that modulate ER stress and apoptosis.

### 2.4. Pharmacological Modulation of eIF2α Phosphorylation

The complex nature of the UPR pathway, having both prosurvival and proapoptotic functions, has made this pathway a popular target for therapeutic intervention. Several clinically significant drugs have been developed that inhibit or activate the PERK pathway, both of which can be cytoprotective in the correct context [108,109,110,111]. Excessive activation of the PERK–eIF2α-P pathway has been implicated in the progression of neurodegeneration in several diseases, including Alzheimer’s disease and Prion infection. In contrast, stalling the UPR in the early phase by inhibiting GADD34 has been cytoprotective in other models, such as amyotrophic lateral sclerosis (ALS) and Charcot–Marie–Tooth disease (CMT). Drugs inhibiting PP1 catalytic activity are of limited utility, because PP1 has too many cellular targets, and because they also inhibit PP2A, resulting in significant toxicity [112,113]. However, multiple small-molecule drugs have been found to inhibit the function of these eIF2ɑ phosphatase-targeting subunits in vivo [111,114,115]. Salubrinal is a drug that specifically blocks the serine/threonine phosphatase-dependent dephosphorylation of eIF2α by inhibiting the PP1 complexes that form with both GADD34 and CReP [116]. Although it acts as an eIF2α phosphatase inhibitor that blocks both GADD34 and CReP function, its precise mechanism of action is not understood. Mice models of ALS, Parkinson’s Disease and α-synucleinopathy show a protective effect of inhibiting eIF2α phosphatase activity by salubrinal treatment [117,118]. This is attributed to the stalling of UPR progression, allowing the clearance of accumulated misfolded ER proteins and potential removal of damaged ER via autophagy. In contrast, prion-mediated neurodegeneration was elevated by salubrinal treatment and reduced with PERK inhibition, strongly suggesting that sustained eIF2α phosphorylation was deleterious in this model [119]. Salubrinal treatment also increases the expression of Aβ in mouse models of Alzheimer’s disease, increasing amyloidogenesis and accelerating Alzheimer’s disease onset and progression [120]. However, salubrinal was still protective against Aβ-mediated cytotoxicity [121]. Sal003 is a salubrinal analog that is more potent and more soluble [122].

Another small-molecule drug, guanabenz, specifically inhibits the dephosphorylation of eIF2α mediated by GADD34, while having no effect on CReP activity [123]. Guanabenz was found to increase cell viability in response to ER stress through its specific interaction with GADD34, leading to enhanced PERK signaling, which resulted in sustained eIF2α phosphorylation and decreased accumulation of ER stress markers, such as BiP, Grp94, ATF4 and CHOP, causing a delay in translational recovery [124]. The optimal dosage of guanabenz needed to produce this cytoprotective effect was less than the amount needed for complete inhibition of GADD34, showing that complete inhibition of GADD34 is detrimental to survival [123]. Guanabenz is also an agonist of the α2-adrenergic receptor, which may limit its utility and has encouraged the search for alternatives [123,125].

A guanabenz isomer, Raphin1 (rational inhibitor of a holophosphatase), was discovered to function as an inhibitor of the CReP–PP1c holoenzyme in both in vitro and in vivo studies [126]. Raphin1 induces a conformational change in CReP, targeting it to the proteasome for degradation in a p97-dependent manner, which compromises substrate recruitment and dephosphorylation [126]. In a mouse model of Huntington’s disease, Raphin1 effectively reduced deficits, was orally bioavailable and crossed the blood–brain barrier [126].

Sephin1 (selective inhibitor of a holophosphatase) is a guanabenz derivative shown to specifically inhibit GADD34 without having measurable α2-adrenergic side effects (in cells or in vivo) [125]. Sephin1 prolongs eIF2α phosphorylation after stress, delaying translation recovery and reducing the expression of the proapoptotic CHOP [125]. In mice, Sephin1 delayed progression and ameliorated two protein-misfolding diseases: Charcot–Marie–Tooth 1B and amyotrophic lateral sclerosis [125]. A potent guanabenz analog, PromISR-6 (promoters of ISR), was found to elicit the integrated stress response, increase eIF2α phosphorylation, prolong translational repression and activate autophagy [127]. This unique ability to stimulate autophagic clearance of intracellular aggregates promoted survival in a cellular model of Huntington’s disease [127].

It should be noted, however, that other studies have challenged these findings regarding guanabenz and Sephin1, with data showing that neither molecule restores proteostasis by interfering with eIF2α-P dephosphorylation [128]. Both Sephin1 and Guanabenz had no effect on the stability of the PP1–GADD34 complex nor substrate-specific dephosphorylation in in vitro enzymatic assays. Sephin1 was inert in a Bio-Layer Interferometry assay that directly measured the affinity between GADD34 and PP1 and also did not interfere with eIF2α-P dephosphorylation in cells in a kinase shut-off experiment. Lastly, Sephin1 was able to reduce the effects of a challenge to proteostasis in cells lacking GADD34, suggesting that Sephin1 disruption of the PP1–GADD34 complex in vivo may be indirect [128]. In contrast, other studies have been able to use reconstituted GADD34 and CReP holoenzyme complexes to demonstrate selective conformational changes in GADD34 following Sephin1 treatment [129]. Further studies are clearly needed to resolve these discrepancies.

In addition to eIF2α-phosphatase inhibitors, several small-molecule inhibitors of PERK have proven neuroprotective despite the opposite effect on UPR signaling. This potentially paradoxical effect has not been fully characterized and may be caused by the differential sensitivity of certain neuronal subtypes to the precise timing of UPR signaling; the disruption of either phosphatase or kinase signaling could be protective in the correct context (reviewed in [130]). Also, the inhibition of PERK can paradoxically activate the ISR through activation of GCN2, further complicating the interpretation of these inhibitors [131]. GlaxoSmithKline(GSK)2606414 is a first-generation PERK inhibitor, with good in vivo and in vitro efficacy [132]. GSK2606414-treated prion-infected mice had reduced PERK activity and eIF2α phosphorylation and increased protein synthesis [133]. In addition, the induction of ATF4 and CHOP was reduced, leading to neuroprotection [133]. Similar neuroprotection occurs with overexpression of GADD34 [119]. In addition, in mouse models of Parkinson’s disease, PERK inhibition is neuroprotective, leading to increased dopamine synthesis and increased expression of synaptic proteins [134]. Inhibition also delayed neurodegeneration in mouse models of Marinesco–Sjogren syndrome, where PERK inhibition not only decreased neuron loss but increased cognitive function and alleviated clinical symptoms [135]. In mouse models of tau-mediated frontotemporal dementia, control animals displayed reduced motility, hunched posture and poor grooming at 8 months, but GSK2606414-treatment mice displayed normal behaviors [136]. Although GSK2606414-treatment was neuroprotective in multiple disorders, significant deleterious side effects were observed, including bodyweight loss and hyperglycemia, which may correlate with pancreatic toxicity [133,134]. Another complication with interpreting PERK inhibition studies is the observation that GSK2606414 and alternative PERK inhibitors also inhibit RIPK, which is a major regulator of apoptotic signaling [137]. Thus, it is unclear to what extent the cytoprotective effects of GSK2606414 are PERK-dependent or RIPK-dependent and if other kinases are indeed involved.

ISRIB is a unique small molecule that blocks the downstream effects of eIF2α phosphorylation, preventing the induction of ATF4, CHOP and GADD34, but it has no effect on PERK or eIF2α phosphorylation [138,139]. ISRIB is neuroprotective in ALS and Aβ models and produces few side effects, including no apparent pancreatic toxicity [140,141]. Despite its efficacy and low toxicity, ISRIB is not considered a promising drug for humans due to low water solubility [109,110].

Overall, multiple steps of the PERK pathway have been evaluated as targets for pharmacological intervention. Drugs targeting GADD34 have shown considerable promise, especially in the potential treatment of neurodegenerative diseases. The exact mechanism of action of these agents, however, is still unclear. A more complete understanding of the molecular mechanisms used by GADD34 and CReP will be necessary to elucidate the precise action of many of these drugs and will aid in the development of additional agents.

## 3. Molecular Properties and Binding Partners of GADD34 and CReP

### 3.1. Biochemistry of eIF2α Phosphatases

**Figure 4:**
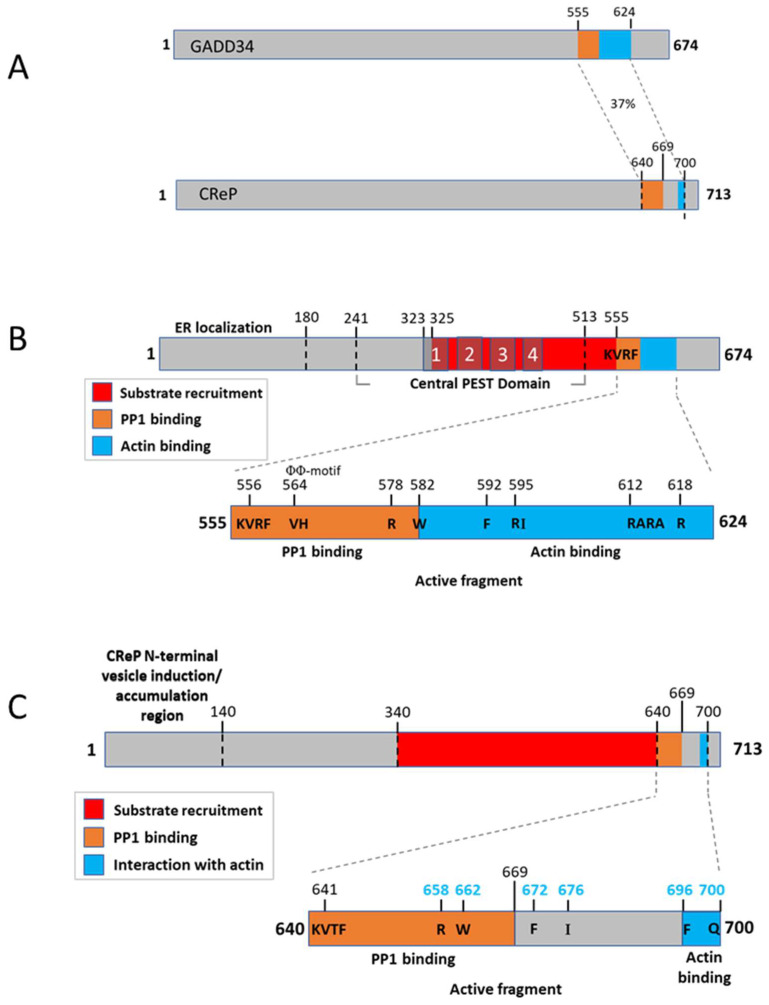
Schematic representation of human GADD34 and CReP: (**A**) Human GADD34 and CReP are 674- and 713-amino-acid-long peptides. The two proteins are 37% identical and 60% similar within the conserved PP1-binding domain in the C-terminus but have little homology outside of this region. PP1 interacting sequences are colored yellow, actin binding domains are colored blue and regions involved in substrate binding are colored red (**B**). The N-terminus of GADD34 contains an ER localization domain. The C-terminus contains the conserved domains required for binding PP1, actin and eIF2α. GADD34 also contains central PEST repeats, which facilitate eIF2α binding. (**C**). The N-terminus of CReP contains an ER localization domain that is not conserved with GADD34. The C-terminus of CReP is highly conserved with GADD34 and contains similar targeting sequences for PP1, actin and eIF2α.

Growth arrest and DNA damage-inducible protein 34 (GADD34) act as a negative feedback inhibitor of UPR signaling through interaction with PP1 to dephosphorylate eIF2α (Figure 4) [22]. The induction of GADD34 expression by ER stress is dependent on the activity of PERK [22]. Human GADD34 is a 674 amino acid, 73.5 kDa, a PP1 regulatory subunit that directs PP1 substrate recognition and localization to the ER following ER stress [24]. GADD34 functions as a scaffold, independently interacting with PP1 and eIF2α [142]. To promote eIF2α dephosphorylation, GADD34 targets PP1 to the ER to form an eIF2α phosphatase complex [24], using an amphipathic helix embedding amino acids methionine 26, serine 30 and alanine 32 in the ER membrane [143]. PP1 binding requires the conserved C-terminal KVRF PP1-binding motif (which binds the hydrophobic RVxF-binding pocket to the surface of PP1 [84,85]), a RARA motif C-terminal to the KVRF motif and ΦΦ motifs [24,142]. The KVRF PP1-binding motif is located at amino acid residues 555–558, and the RARA motif is located at residues 612–615, within a region that contains several arginine–alanine repeats conserved in mammalian and viral GADD34-like proteins [24,144,145]. Arginine-618 was found to specifically strengthen PP1 binding [24]. The ΦΦ motif is formed by Val564 and His565, with His565 forming a π-stacking interaction with Tyr78 in PP1 [142]. The canonical RVxF motif is found in ≥85% of PP1 regulators, and the ΦΦ motif is predicted to be present in ~20% of PP1 regulators [142,146]. The association of eIF2α with GADD34 is independent of PP1 binding, with the central PEST domain (residues 241–513) serving as the primary, independent and high-affinity binding site for eIF2α on GADD34 [142,147] and is required for stable eIF2α recruitment [142]. The cofactor G actin is important for both stabilizing the PPP1R15–PP1 complex and allowing for eIF2α substrate specificity [148]. Arginine 578 engages with a surface arginine pocket of PP1 and plays an important role, along with tryptophan residue 582, in allowing G-actin to activate substrate-specific dephosphorylation and thus attenuate the UPR [149]. Two other conserved residues, phenylalanine 592 and isoleucine 596, were also found to play a role in recruiting G-actin to the holophosphatase complex, although to a lesser degree [148]. F592 inserts into a hydrophobic cavity on the surface of actin, Arg595 forms a salt bridge with actin Asp334 and Arg612 forms hydrogen bonds with actin Tyr169 and Met355 [149]. Finally, the five C-terminal residues of the functional core of GADD34 (W616-R620) have been found to be essential for actin association and are predicted to form a short amphipathic helical region [148,150]. ER localization is determined by the N-terminal 180 amino acids of GADD34 [24] and is mediated by the hydrophobic surface of an N-terminal amphipathic helix, specifically valine 25 and leucine 29 [143].

A decline in eIF2α phosphorylation is seen in response to ER stress prior to GADD34 protein detection, which indicates that another mechanism is at work to initially contribute to this decline. CReP, named “Constitutive Repressor of eIF2α Phosphorylation”, was found to have homology in the C-terminal end to GADD34 and could also bind PP1 (Figure 4). It was given its name due to its presence in unstressed cells and its primary function of regulating basal levels of eIF2α phosphorylation [23]. CReP is a 713 amino acid, 79.2 kDa protein that recruits PP1 to dephosphorylate eIF2α. The recruitment of the substrate, eIF2α, is encoded by the conserved region 340–639; this region was found to be necessary and sufficient for eIF2α recruitment [151,152,153]. The conserved COOH-terminal functional core of CReP specifies interaction with PP1, recruits the essential cofactor G-actin and allows for substrate-specific dephosphorylation [148,150]. CReP contains the RVxF PP1-binding motif (amino acid residues 640–643) required for the formation of the CReP–PP1 complex to dephosphorylate eIF2α [147]. The side chains of V641 and F643 in this motif engage hydrophobic crevices on the back of PP1 [142]. Unlike GADD34, PEST repeats are not present in CReP. The five C-terminal residues of the functional core of CReP (F696-Q700) are predicted to form a short amphipathic helical region involved in actin association [148]. Arginine 658 engages with a deep pocket on the surface of PP1 and allows for an ionic interaction with PP1 residue D71, playing a critical role in PP1 binding by CReP [148]. This residue (R658) also seems to play an important role in allowing actin to activate substrate-specific dephosphorylation [148]. Three other conserved residues of CReP (tryptophan 662, phenylalanine 672 and isoleucine 676) were also found to play a role in G-actin recruitment to the holophosphatase complex but to a lesser degree than R658 [148]. The N-terminal amino acids 1–140 of CReP contain an amphipathic α-helix domain that is both necessary and sufficient for the ability of CReP to interact with membranes and confer vesicle association and has been termed the “CReP N-terminal vesicle induction/accumulation region” [143,151]. The discovery that CReP plays a role in regulating the uptake of Staphylococcus aureus α-toxin by epithelial cells underlies PP1c-independent roles for CReP in the cell, namely in membrane trafficking [151].

### 3.2. Transcriptional and Translational Regulation of GADD34 and CReP

Stress-induced expression of GADD34 is perhaps the most frequently observed mechanism for regulating eIF2α dephosphorylation. GADD34 was first identified as a stress-induced gene [154] and is upregulated by a variety of cellular stresses, including ionizing radiation [155], UV radiation [156], ER stress [22], amino acid starvation [22], apoptosis [157,158] and oxidative stress [79,159]. GADD34 stress-induced upregulation is independent of p53 [154] but frequently dependent on eIF2α kinases and ATF4 [22]. In contrast to amino acid starvation or ER stress, GADD34 upregulation after oxidative stress is independent of eIF2α phosphorylation and ATF4 [160]. The stress induction of GADD34 plays a critical role in the feedback inhibition of eIF2α phosphorylation, allowing a transition from an early phase of UPR signaling characterized by translational repression to a late phase with restored translation [22,28,89].

In contrast, CReP was originally identified as a constitutively expressed eIF2α phosphatase, primarily responsible for basal dephosphorylation, since it was not transcriptionally upregulated by the ER or oxidative stressors tunicamycin or arsenite [23]. However, additional studies have clearly shown that under certain circumstances, CReP can be dynamically expressed and that GADD34 and CReP have strongly overlapping roles in the kinetics of UPR signaling. In the absence of CReP, the basal levels of phosphorylated eIF2α are higher and have a delayed recovery from ER stress, showing that CReP plays a role in the ER stress response [23]. Also, despite its low expression in unstressed cells, GADD34 plays a critical role in the basal dephosphorylation of eIF2α and the early UPR [28]. CReP upregulation can compensate for a lack of GADD34: CReP mRNA showed a significant 10-fold increase in GADD34 KO cells compared with WT over 16 h post stress induction, suggesting that a lack of GADD34 results in the enhanced transcription of the CReP gene or stabilization of CReP mRNA in response to stress [28]. In addition, human patients with CReP mutations have compensatory increases in GADD34 expression [92].

Similar to canonical stress-inducible genes such as ATF4 and CHOP [161], both GADD34 and CReP contain uORFs, which allow for more efficient translation in the presence of eIF2α phosphorylation [162]. These uORFs are evolutionarily conserved and allow the stress induction of dPPP1R15 in Drosophila, even though the homolog is not transcriptionally upregulated [87]. In addition, both GADD34 and CReP proteins are short-lived, consistent with being dynamically expressed proteins [23,159]. CReP degradation mediated by VCP/p97 regulates the integrated stress response [163], and DNA damage signaling regulates eIF2α phosphorylation by promoting CReP degradation through the F-box protein βTRCP [164]. CReP mRNA is also one of the main substrates of IRE1α-dependent decay during UPR activation, which contributes to elevating the levels of phosphorylated eIF2α independent of PERK kinase activity [165]. Also, CReP is targeted by mir-98-5p, providing additional mechanisms to control CReP expression [166,167]. These findings suggest that the eIF2α phosphatases may be coordinately regulated to manage phosphorylated eIF2α levels during ER stress.

### 3.3. GADD34 and CReP Binding Partners and Posttranslational Regulation

The posttranslational regulation of GADD34 and CReP has been less well studied than transcriptional and translational control. GADD34 and CReP are both highly phosphorylated proteins [168]. The specific function of each phosphorylation site has been poorly characterized. GADD34 is also acetylated, but the significance of this modification is unexamined [169]. The phosphorylation of tyrosine 262 of human GADD34 promotes proteolytic degradation, and this site is dephosphorylated by TC-PTP (PTPN2) [168]. The mutation of a large number of phosphorylation sites in CReP renders the protein resistant to protein turnover [164].

A number of proteins have been identified as GADD34 or CReP binding partners using proteomics or yeast two-hybrid approaches (the published GADD34 and CReP binding partners are listed in Table S1). As discussed in the previous sections, some binding partners are essential components of the active eIF2α complex, such as PP1, G-actin and eIF2α. G-actin was identified as a conserved GADD34 and CReP binding partner using a proteomics approach, which also identified a number of other binding partners, including tubulin and heat shock proteins [150]. Several proteins have been identified that can bind to GADD34 and inhibit PP1 catalytic activity. Protein phosphatase inhibitor-1 (I-1 or PPP1R1A) was identified as a GADD34 binding partner using yeast two-hybrid analysis and was confirmed by pulldown and co-IP experiments [15]. I-1 binds within the unique central region of GADD34 and forms a stable three-part complex with GADD34 and PP1 [15]. When phosphorylated by PKA, I-1 can inhibit PP1 within the three-part complex in vitro [15] and can increase eIF2α phosphorylation in mammalian cells [170]. Bag-1 also binds to GADD34 and negatively regulates its activity. Bag-1 was identified as a GADD34 binding protein and confirmed by co-immunoprecipitation [171]. Bag-1 binds to GADD34 and recruits Hsp70 to the complex [171]. PP1 catalytic activity is inhibited by Bag-1 binding, and the coexpression of Bag-1 blocks the ability of GADD34 to arrest cell proliferation in colony formation assays [171].

Several proteins have been identified as GADD34 binding partners that can modulate the ability of GADD34 to promote apoptosis when overexpressed. Whether these proteins function in an eIF2α-dependent or -independent manner is unclear. The human trithorax leukemia fusion protein (HRX) interacts with GADD34 and forms a stable complex with GADD34 and the chromatic remodeler SNF5/INI1 [172,173]. HRX proteins block the proapoptotic effects of GADD34 overexpression [172], while SNF4/INI1 can form a stable active complex with GADD34 and PP1 [173]. The Src-family tyrosine kinase Lyn can also bind to GADD34 and block its proapoptotic activity [174].

GADD34 binds to the NAD-dependent protein deacetylase SIRT1 and dephosphorylates serine 47, activating SIRT1 [169]. Activated SIRT1 then deacetylates eIF2α [175], which promotes dephosphorylation by GADD34/PP1 [169]. The knockdown of SIRT1 or GADD34 promotes eIF2α phosphorylation and stress-induced apoptosis [169].

Several proteins have been identified as GADD34 binding proteins, but their in vivo function is unclear. The kinesin KIF3A was identified as a GADD34 binding partner by yeast two-hybrid approaches and confirmed by in vitro pulldown experiments [176]. Hsp40 was similarly identified [177]. Both KIF3A and Hsp40 bind primarily to the C-terminal PP1-binding domain, but GADD34 PEST repeats were required for strong binding [176,177]. Similarly, yeast two-hybrid approaches identified the recombination protein Translin [178] and the uncharacterized protein G34BP [179]. GADD34 also interacts with the zinc-finger transcription factor BFCOL1 [180]. Proteomics approaches have identified a large number of putative GADD34 binding partners or substrates [150,181]. A few targets, such as the COP9 signalosome subunit COPS5, WD repeat-containing protein WDR5 and the gradual stress proteins CAPRIN1 and G3BP1, were validated as binding partners, and COPS5 dephosphorylation was dependent on GADD34 activity [181].

GADD34 has also been identified as a binding partner for several proteins regulating pathways independent of the PERK pathway. Chronic oxidative stress induced by arsenite promotes the GADD34-mediated recruitment of casein kinase-1ɛ (CK1ɛ), allowing it to phosphorylate the TAR DNA-binding protein TDP-43 [160]. The increased phosphorylation of TDP-43 potentially contributes to the pathobiology of ALS and other neurodegenerative diseases [160]. GADD34 also interacts with Smad7 to promote the dephosphorylation of type I TGF beta receptors [182] and CUEDC2 (CUE domain-containing protein 2) and recruits PP1 to inactive IKK [183].

A lot of studies have focused on the regulation of PP1 by GADD34 and CReP, with a detailed understanding of how this interaction occurs. Though only 37% identical within the PP1-binding domain, both proteins seem to work in a very similar way and share many of the same additional binding partners (such as actin). However, the function of many of the other potential protein domains found within GADD34 and CReP have been less studied, and they show less similarity in the sequencing and conservation of amino acid sequences. The transcriptional and translational regulation of GADD34 has been well-studied, but less is known about CReP, likely because it was considered to be constitutive. A large number of protein-binding partners have been identified for both GADD34 and CReP; however, the physiological significance of the majority of them remains unclear.

## 4. Diverse Cellular Functions of GADD34 and CReP

While genetic studies in mice have shown that the primary cellular role of GADD34 and CReP is to dephosphorylate eIF2α [88], GADD34 and CReP have been clearly implicated in other signaling pathways. The mammalian homolog of TOR (mTOR) is a conserved protein serine/threonine kinase and plays a prominent role in cell growth, proliferation, motility, survival, gene transcription and protein synthesis in response to hormones, growth factors and nutrients [184]. While GADD34 and mTOR are generally considered to be independently regulated, GADD34 expression and mTOR signaling are inversely correlated during cellular responses to DNA damage, bioenergetic starvation or hypoxia [185]. GADD34 also protects cells from apoptosis caused by glucose depletion via mTOR inhibition [186]. Starvation induces GADD34 to form a stable complex with Tuberous sclerosis complex 1 (TSC1) and Tuberous sclerosis complex 2 (TSC2), which suppresses the mTOR signaling pathway and induces autophagy [187].

GADD34 also regulates TGFβ signaling [182]. TGFβ superfamily members regulate cell fate by controlling cell proliferation, cell differentiation and apoptosis [188]. Poorly regulated TGFβ family signaling has been shown in various human diseases, such as autoimmune diseases, vascular disorders and cancer [189]. These proteins elicit cellular responses through the ligand-induced formation of heteromeric complexes of specific transmembrane kinase receptors (type I and type II). A type II receptor is a constitutively active kinase, which phosphorylates particular serine and threonine residues in type I receptors, leading to their activation [188]. This signaling pathway’s activity is tightly regulated by serine/threonine phosphorylation, which plays a key role in controlling protein–protein interactions in signaling responses [190]. Smad7, an inhibitory Smad protein whose expression is induced by TGFβ, interacts with GADD34 to stimulate the dephosphorylation (via PP1) of the TGFβ type 1 receptor, functioning as a negative feedback mechanism in the TGFβ signaling pathway [182]. As more studies are conducted on GADD34 and CReP function, we will likely find new roles for these targeting subunits in other cellular processes.

## 5. Future Directions

Since their initial discovery, a large body of work has been generated on the eIF2α phosphatase-targeting subunits, with much more attention given to GADD34 than CReP, likely due to the initial impression that GADD34 had more complex regulation, while CReP was a constitutive gene. Several open questions remain about the function of these proteins. First, the degree of genetic redundancy between GADD34 and CReP needs to be further examined. Are the differing phenotypes for GADD34 and CReP produced by genetic knockout or pharmacological inhibition caused by a difference in the biochemical function of the protein products or are they better explained by temporal differences in gene expression? Secondly, it remains controversial whether drugs such as Sephin1 work in a GADD34-dependent or independent manner. Further work is needed to clarify the mechanism of action of these pharmacological agents. Thirdly, more work will be needed to understand the context-dependency of GADD34 knockdown on cell survival. Why is GADD34 inhibition protective in some cellular contexts and deleterious in others? Additionally, GADD34 and CReP are known to bind a large number of other proteins in addition to eIF2α, PP1 and actin, but the physiological significance of only a few of these binding partners has thoroughly been characterized. Finally, both GADD34 and CReP are heavily posttranslationally modified, but the significance of most of these modifications on protein function and turnover is unclear. Given the large number of pharmacological agents in development that target the UPR, these questions will be of considerable interest.

## Supplementary Materials

The following supporting information can be downloaded at https://www.mdpi.com/article/10.3390/ijms242417321/s1.
