# Supplementary material for: The PPP1R15 Family of eIF2-alpha Phosphatase Targeting Subunits (GADD34 and CReP)
Source: Int J Mol Sci. 2023 Dec 10;24(24):17321. doi: 10.3390/ijms242417321 (PMC10743859; doi:10.3390/ijms242417321)
Supplement: Supplementary file 1 [file ijms-24-17321-s001.zip › ijms-2715297-supplementary.pdf]

GADD34 Binding Proteins

Protein	Identification Technique	Reference
PP1CA	Affinity purification; Mass Spectrometry	150
PP1CB	Affinity purification; Mass Spectrometry	150
PP1CC	Affinity purification; Mass Spectrometry	150
HSPA8	Affinity purification; Mass Spectrometry	150
HSPA1A	Affinity purification; Mass Spectrometry	150
HSPA2	Affinity purification; Mass Spectrometry	150
HSPA6	Affinity purification; Mass Spectrometry	150
ACTB	Affinity purification; Mass Spectrometry	150
ACTG1	Affinity purification; Mass Spectrometry	150
ACTBL2	Affinity purification; Mass Spectrometry	150
ACTA2	Affinity purification; Mass Spectrometry	150
TUBB	Affinity purification; Mass Spectrometry	150
TUBB4B	Affinity purification; Mass Spectrometry	150
TUBB2A	Affinity purification; Mass Spectrometry	150
TUBB6	Affinity purification; Mass Spectrometry	150
TUBB4A	Affinity purification; Mass Spectrometry	150
TUBA1C	Affinity purification; Mass Spectrometry	150
TUBA1B	Affinity purification; Mass Spectrometry	150
TUBA4A	Affinity purification; Mass Spectrometry	150
HADHB	Affinity purification; Mass Spectrometry	150
HADHA	Affinity purification; Mass Spectrometry	150
PRKDC	Affinity purification; Mass Spectrometry	150
EIF2S1	Affinity purification; Mass Spectrometry	150
EIF2S2	Affinity purification; Mass Spectrometry	150
EIF2S3	Affinity purification; Mass Spectrometry	150
InsIRS4	Affinity purification; Mass Spectrometry	150
DYNC1H1	Affinity purification; Mass Spectrometry	150
EEF1A1	Affinity purification; Mass Spectrometry	150
AIFM1	Affinity purification; Mass Spectrometry	150
CAD	Affinity purification; Mass Spectrometry	150
SMC2	Affinity purification; Mass Spectrometry	150
DNAJA1	Affinity purification; Mass Spectrometry	150
TCP1	Affinity purification; Mass Spectrometry	150
TCP1beta	Affinity purification; Mass Spectrometry	150
Translin	Yeast two-hybrid; In vitro binding assay	178
Leukemic HRX Proteins -HRX-ENL; HRX-AF9; HRX-ELL	Yeast two hybrid; co-immunoprecipitation	172
Human SNF5/INI1 protein	Co-immunoprecipitation assay; in vitro binding assay	172
KIF3A (part of the Kinesin superfamily)	Yeast two-hybrid assay; In vitro binding assay	176
Src-related protein tyrosine kinase Lyn	Yeast two-hybrid; co-immunoprecipitation	174
Inhibitor 1 (ppp1r1a)	Yeast two-hybrid; co-immunoprecipitation	15
BFCOL1	Yeast two-hybrid; In vitro binding assay	180
Smad7	Yeast two-hybrid; co-immunoprecipitation	182
PTPN2	Mass-spectrometry; substrate-trapping studies	168
CUEDC2 (CUE domain–containing protein 2)	Yeast two-hybrid; co-immunoprecipitation	183
casein kinase-1ε (CK1ε)	co-immunoprecipitation	160
TDP-43	co-immunoprecipitation	160
SIRT1	co-immunoprecipitation	169
COPS5	Affinity purification; Mass Spectrometry	181
WDR95	Affinity purification; Mass Spectrometry	181
Caprin1	Affinity purification; Mass Spectrometry	181
G3BP1	Affinity purification; Mass Spectrometry	181
Hsp40	Yeast two hybrid; co-immunoprecipitation	177
Bag1	Yeast two hybrid; co-immunoprecipitation	171
G34BP	Yeast two hybrid; co-immunoprecipitation	179
TSC1/TSC2	Co-immunoprecipitation	186

CReP Binding Proteins

Protein	Identification Technique	Reference
PP1CA	Affinity purification; Mass Spectrometry	150
PP1CB	Affinity purification; Mass Spectrometry	150
PP1CC	Affinity purification; Mass Spectrometry	150
HSPA8	Affinity purification; Mass Spectrometry	150
HSPA1A	Affinity purification; Mass Spectrometry	150
HSPA2	Affinity purification; Mass Spectrometry	150
HSPA6	Affinity purification; Mass Spectrometry	150
ACTB	Affinity purification; Mass Spectrometry	150
ACTG1	Affinity purification; Mass Spectrometry	150
ACTBL2	Affinity purification; Mass Spectrometry	150
ACTA2	Affinity purification; Mass Spectrometry	150
TUBB	Affinity purification; Mass Spectrometry	150
TUBB4B	Affinity purification; Mass Spectrometry	150
TUBB2A	Affinity purification; Mass Spectrometry	150
TUBB6	Affinity purification; Mass Spectrometry	150
TUBB4A	Affinity purification; Mass Spectrometry	150
TUBA1C	Affinity purification; Mass Spectrometry	150
TUBA1B	Affinity purification; Mass Spectrometry	150
TUBA4A	Affinity purification; Mass Spectrometry	150
HADHB	Affinity purification; Mass Spectrometry	150
HADHA	Affinity purification; Mass Spectrometry	150
PRKDC	Affinity purification; Mass Spectrometry	150
EIF2S1	Affinity purification; Mass Spectrometry	150
EIF2S2	Affinity purification; Mass Spectrometry	150
EIF2S3	Affinity purification; Mass Spectrometry	150
InsIRS4	Affinity purification; Mass Spectrometry	150
DYNC1H1	Affinity purification; Mass Spectrometry	150
EEF1A1	Affinity purification; Mass Spectrometry	150
